# Stretchable Dual-Capacitor Multi-Sensor for Touch-Curvature-Pressure-Strain Sensing

**DOI:** 10.1038/s41598-017-11217-w

**Published:** 2017-09-07

**Authors:** Hanbyul Jin, Sungchul Jung, Junhyung Kim, Sanghyun Heo, Jaeik Lim, Wonsang Park, Hye Yong Chu, Franklin Bien, Kibog Park

**Affiliations:** 1 School of Electrical and Computer Engineering, Ulsan National Institute of Science and Technology (UNIST), Ulsan, 44919 Republic of Korea; 2 Department of Physics, Ulsan National Institute of Science and Technology (UNIST), Ulsan, 44919 Republic of Korea; 3 Samsung Display Giheung Campus, Yongin-si, Gyeonggi-do 17113 Republic of Korea

## Abstract

We introduce a new type of multi-functional capacitive sensor that can sense several different external stimuli. It is fabricated only with polydimethylsiloxane (PDMS) films and silver nanowire electrodes by using selective oxygen plasma treatment method without photolithography and etching processes. Differently from the conventional single-capacitor multi-functional sensors, our new multi-functional sensor is composed of two vertically-stacked capacitors (dual-capacitor). The unique dual-capacitor structure can detect the type and strength of external stimuli including curvature, pressure, strain, and touch with clear distinction, and it can also detect the surface-normal directionality of curvature, pressure, and touch. Meanwhile, the conventional single-capacitor sensor has ambiguity in distinguishing curvature and pressure and it can detect only the strength of external stimulus. The type, directionality, and strength of external stimulus can be determined based on the relative capacitance changes of the two stacked capacitors. Additionally, the logical flow reflected on a tree structure with its branches reaching the direction and strength of the corresponding external stimulus unambiguously is devised. This logical flow can be readily implemented in the sensor driving circuit if the dual-capacitor sensor is commercialized actually in the future.

## Introduction

Due to the increasing demand for human-machine interface devices used in robotics^1–3^, internet of things (IoT)^4, 5^, and personal healthcare monitoring systems^6–10^, the performances and functionalities of associated sensors are required to be enhanced accordingly^11–18^. In human-machine interface systems, sensors play crucial roles in conceiving signals from human, such as bioelectrical signals in electroencephalogram (EEG)^19–23^ and electrocardiogram (ECG)^23–26^ and body motions^11, 27–33^, and then delivering them to machine correctly. With the aids of sensors, a machine can acquire several sensing capabilities like human and also be controlled properly by the conceived signals^34^. For sophisticated sensing, sensors should be able to detect and distinguish several different signals (Multi-functionality). Also, stretchability is an essential property needed especially for attaching sensors to skin or clothes^35–40^.

Recently, many groups have studied skin-mountable multi-functional capacitive sensors by using various materials. The Zhu’s group reported a wearable multi-functional sensor based on silver nanowire (AgNW), which could detect strain, pressure, finger touch, and various human motions such as walking, running, squatting, and jumping^16^. The Lacour’s group also introduced a stretchable multi-functional sensor made of gold thin films embedded in polydimethylsiloxane (PDMS) which could detect strain, pressure, and finger touch^14^. However, all former researches are based on single-capacitor sensors which have some inevitable limitations. Firstly, they can detect only the magnitude of stimulus with no information about direction. Secondly, it is not possible to distinguish the types of detected stimuli quite clearly just based on the capacitance change of single capacitor. For example, single-capacitor sensors show the similar capacitance increase for pressure and strain applied on them. In this work, we introduce a new type of dual-capacitor multi-functional sensor to overcome the limitations of the sensors developed so far. Unlike the aforementioned single-capacitor sensor, our dual-capacitor sensor consists of two vertically stacked capacitors in each pixel so that it can classify the type of external stimulus unambiguously and also can detect its strength and surface-normal directionality. These features of our dual-capacitor sensor are confirmed for curvature, pressure, touch, and strain sensing.

## Results

### Non-lithographic fabrication processes of dual-capacitor sensor

**Figure 1:**
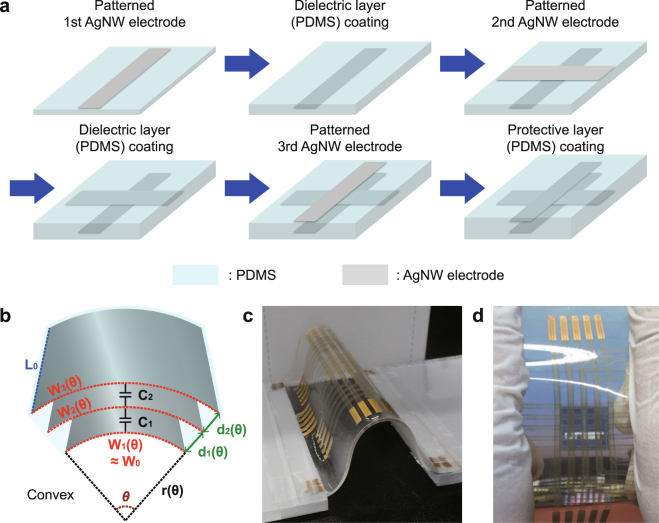
Stretchable dual-capacitor sensor composed of AgNW electrodes and PDMS insulators. (**a**) Schematic view of the fabrication processes of dual-capacitor sensor which has two capacitors stacked vertically. (**b**) The schematic view of dual-capacitor sensor during curvature sensing when the sensor is in convex shape with the curvature radius of *r*. Photographic image of 5 × 5 array type dual-capacitor sensor (**c**) bent in convex shape and (**d**) stretched by hand.

Figure 1a illustrates the fabrication processes of dual-capacitor multi-functional sensor in 5 × 5 array form. More detailed descriptions for the fabrication processes can be found in experimental section. Basically, the whole sensor was fabricated just by using AgNW and PDMS. The substrate, lower dielectric layer, upper dielectric layer, and protective layer were formed with PDMS films while all the electrodes including bottom, middle, and top ones were made by patterning spin-coated AgNW films. Those three electrodes separated from one another by dielectric layers were patterned by using selective oxygen (O_2_) plasma treatment method (See Supplementary Information Fig. S1) to have their line-width of ~2 mm and line-spacing also of ~2 mm^41^. Our selective oxygen plasma treatment method has uniqueness in the sense that it enables patterning AgNW electrodes without using photolithography and etching processes. The thickness of lower and upper insulating layers was ~400 μm commonly. Differently from the previous studies where two PDMS films with line-pattern AgNW electrodes on their surfaces were attached physically to each other for forming a pixelated single-capacitor sensor^11, 16, 17^, all the layers of our dual-capacitor sensor were deposited sequentially, starting from the substrate, which makes it easy to control the thickness of PDMS film and align the two stacked capacitors precisely, and is also advantageous to secure the solid adhesion between stacked layers. The sequential stacking of PDMS layers was achieved by spin-coating liquid PDMS on the surface of hardened underlying PDMS layer with patterned AgNW electrodes and curing it in an oven. The details of curing process are described in “Methods” section below. After curing, the liquid PDMS was found to be firmly bonded to the underlying PDMS layer, implying the excellent adhesion between the PDMS layers at the interface. The cross-sectional optical microscope image of strongly bonded PDMS stacks is shown in Fig. S3 of Supplementary Information. The average measured capacitance of each capacitor in the whole 25 pixels was ~2.2 pF. Figure 1b shows the schematic view of completed dual-capacitor sensor when it is bent into convex with the curvature radius of *r*(*θ*). *θ* is the corresponding curvature angle. Figure 1c and d are the photographic images of the completed sensor, demonstrating its flexible and stretchable properties.

### Curvature sensing

Initially, the lateral sizes (*L*_0_ and *W*_0_) and dielectric layer thicknesses (*d*_0_) of the two parallel-plate capacitors of dual-capacitor sensor are the same as each other. When the dual-capacitor is curved as shown in Fig. 1b (convex), the top electrode (*W*_3_(*θ*)) will be stretched more than the middle electrode (*W*_2_(*θ*)). Meanwhile, the bottom electrode will have almost no stretching (*W*_1_(*θ*) ≈ *W*_0_). Here, it is noted that the PDMS elastomer with its Poisson ratio of nearly 0.5 is known to be incompressible for plastic deformation at relatively small strain and hence the bottom electrode will not be compressed with the plastic bending for curvature sensing^14, 42, 43^. By considering the formula of cylindrical capacitor, the capacitances of lower (*C*_1_) and upper (*C*_2_) capacitors can be represented as the following relations.

1 $$\begin{array}{l}{C}_{1}(\theta )=\frac{2{\varepsilon }_{r}{\varepsilon }_{0}{L}_{0}{W}_{0}}{\mathrm{ln}[1+2{d}_{0}/r(\theta )]}\times \frac{1}{r(\theta )}\\ {C}_{2}(\theta )=\frac{2{\varepsilon }_{r}{\varepsilon }_{0}{L}_{0}{W}_{0}}{\mathrm{ln}[1+2{d}_{0}/\{r(\theta )+2{d}_{0}\}]}\times \frac{1}{r(\theta )}\end{array}$$

**Figure 2:**
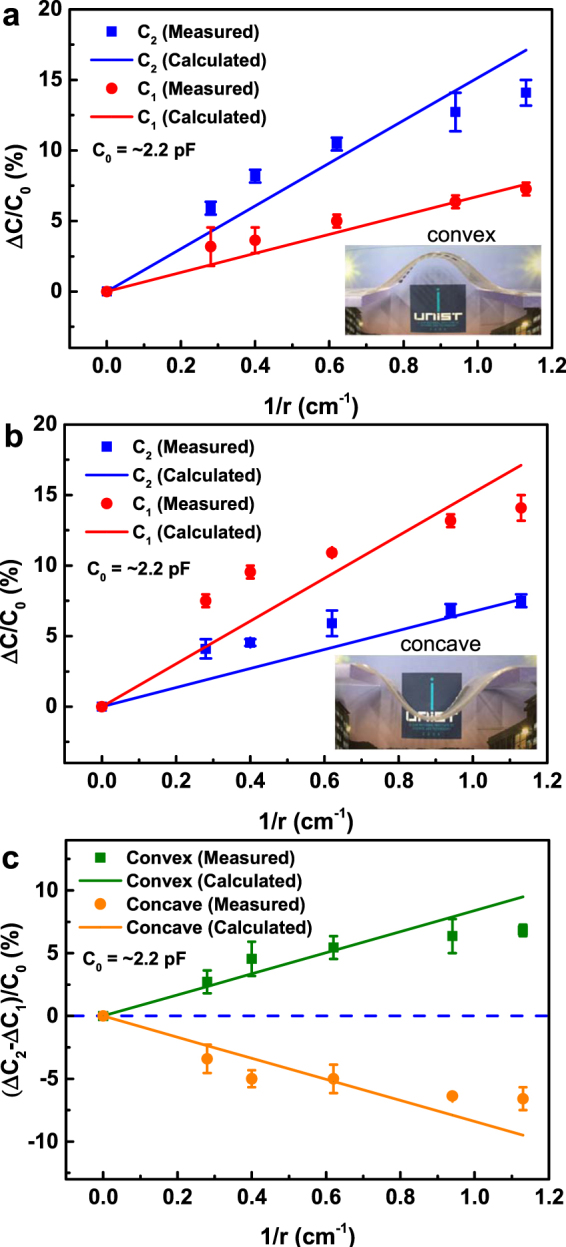
Curvature sensing data for dual-capacitor sensor. The upper (Δ*C*_2_) and lower (Δ*C*_1_) capacitance changes as functions of curvature 1/*r* in (**a**) concave and (**b**) convex shape. (**c**) The difference of Δ*C*_2_ and Δ*C*_1_ versus 1/*r* for both convex and concave shape. Here, the square and circular dots are the measured data and the solid lines represent the theoretical calculations.

Here, *ε*_0_ is vacuum permittivity and *ε*_*r*_ the dielectric constant of dielectric layer. If the dual-capacitor is curved to be concave, the same form of relations will be obtained but this time, *C*_1_ and *C*_2_ will be exchanged mutually. The detailed derivation procedures of equation (1) are described in “Methods” section. Figure 2a and b show the capacitance changes of the lower (Δ*C*_1_) and upper (Δ*C*_2_) capacitors as functions of the curvature 1/*r*. As predicted in equation (1) and the associated statements, *C*_1_ and *C*_2_ increase monotonically. In case of the convex shape (Fig. 2a), Δ*C*_2_ is larger than Δ*C*_1_ for the entire curvature range. On the other hand, Δ*C*_2_ is smaller than Δ*C*_1_ for the concave shape (Fig. 2b). In Fig. 2c, the difference between Δ*C*_1_ and Δ*C*_2_ is plotted also as a function of 1/*r* where the difference is found to increase with the curvature increasing. From these measured data, it is clear that our dual-capacitor sensor can detect the surface-normal directionality and radius of curvature by comparing Δ*C*_1_ and Δ*C*_2_ with proper calibration.

### Pressure sensing

**Figure 3:**
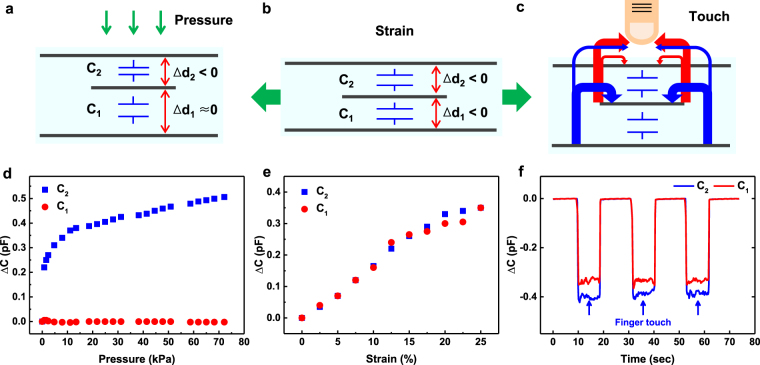
Pressure, strain, and touch sensing data for dual-capacitor sensor. Schematic views showing the operation principles of dual-capacitor sensor for (**a**) pressure, (**b**) strain, and (**c**) touch sensing. The upper (Δ*C*_2_) and lower (Δ*C*_1_) capacitance changes as functions of applied (**d**) pressure, (**e**) strain, and (**f**) repeated finger touch motions.

Similarly to the curvature sensing, our dual-capacitor sensor can conceive both the magnitude and the surface-normal directionality of pressure given to it by measuring Δ*C*_1_ and Δ*C*_2_. Again, in order to determine the magnitude of pressure correctly, the proper calibration connecting the measured capacitance change and the magnitude of pressure needs to be done. When the dual-capacitor sensor is pressed locally with a certain magnitude of force from either upper or lower side (let’s say from the upper side for the following discussion), the applied force will be dispersed sideway more and more as going down through the insulator layer of upper capacitor. Accordingly, the pressure defined as force divided by area will become smaller and smaller as going into the insulator layer. If the insulator of upper capacitor is thick enough, the pressure at the depth of lower capacitor will be reduced significantly. Then, only the insulator of upper capacitor will be compressed while that of lower capacitor is hardly compressed. So to speak, the applied pressure is absorbed almost entirely by the capacitor on the side of the pressure being applied. More concretely, the ~400 μm thick PDMS layer is found to absorb the pressure completely up to ~75 kPa which is the largest pressure used in our experiments. Consequently, only one of the two capacitors will be compressed and its capacitance will increase while the other will hardly change in its shape and capacitance as shown in Fig. 3a. Figure 3d shows the capacitance changes of each capacitor in the dual-capacitor sensor as a function of applied pressure. As expected, only Δ*C*_2_ increases with almost no change of Δ*C*_1_ when the pressure is applied onto the upper side of sensor. For the pressures below 10 kPa, Δ*C*_2_ increases somewhat rapidly and the increase slows down as the pressure becomes further larger. The pressure sensitivity (*S*) is defined to be the slope of the curve plotting the capacitance change ratio depending on pressure as shown below^44^.

2 $$S=\frac{\delta ({\rm{\Delta }}C/{C}_{0})}{\delta p}$$

Here, *p* is the applied pressure, *C*_0_ the initial capacitance with no pressure applied, and Δ*C* the capacitance change referenced from *C*_0_. For the pressures above 10 kPa, Δ*C*_2_ increases more gradually in a linear fashion. The pressure sensitivity in this regime is estimated to be ~1 MPa^−1^ which is higher than other studies using PDMS as an insulating layer^14^. It is considered that the very thin protective layer is the main contributor for high pressure sensitivity which can transfer the applied pressure to the buried capacitor with minimal loss.

### Strain sensing

When the dual-capacitor sensor is stretched out sideway, the thicknesses of two insulating layers will decrease ideally by the same amount and both capacitances, *C*_1_ and *C*_2_, will increase accordingly (Fig. 3b). Figure 3e shows Δ*C*_1_ and Δ*C*_2_ as the strain of sensor increases. Both of them are almost identical to each other and increases linearly up to the strain of 25%. The stability of our dual-capacitor sensor for multiple stretching was confirmed by the stretching and releasing cycle test increased up to 1000 repetitions as shown in Fig. S4. The capacitance of dual-capacitor sensor returned back to its initial value without residual strain after being released from stretching.

### Touch sensing

In case of touch sensing, the redistribution of fringing electric field lines causes the change of measured capacitance^45^, unlike curvature, pressure, and strain sensing for which the capacitance change occurs due to structural deformation. When a finger (or a grounded conductive object) touches the top surface of sensor, some of the fringing electric field lines residing in the insulating layers of two capacitors will be absorbed by the finger. The decreases of fringing electric field lines in the insulating layers are equivalent to reducing the dielectric constants of the insulating layers effectively, resulting in the decreases of measured capacitances. Figure 3c illustrates the distribution of fringing electric field lines with the finger touch on the top surface of sensor. As shown in the figure, the upper capacitor right near the finger will lose more fringing electric field lines to the finger than the lower one. Hence, the capacitance of the upper capacitor will decrease more. These expectations match exactly with the actual measurements (Fig. 3f). When the finger touches on the top side of sensor, Δ*C*_2_ is ~19.0% of the capacitance without touch, larger than Δ*C*_1_ of ~15.5%. These measured touch sensitivities are better than other reported values and we believe that the high touch sensitivities are also due to the relatively thin protective layer in comparison with other studies^16, 17^. The distance between touching finger and buried sensor becomes shorter as the protective layer gets thinner so that more fringing electric fields can be absorbed by the finger, leading to larger capacitance decrease and higher touch sensitivity eventually.

### Unambiguous classification of the external stimuli given to dual-capacitor sensor

**Figure 4:**
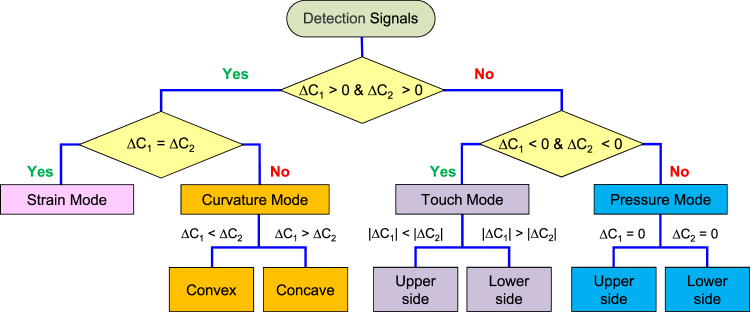
Logical flow for classifying the external stimulus. By comparing the measured capacitance changes of the two capacitors of dual-capacitor sensor carefully, an external stimulus can be classified into curvature, pressure, strain, or touch unambiguously.

So far, we have demonstrated that our dual-capacitor sensor can detect curvature, pressure, strain, and touch altogether. Then, one question raised naturally is whether or not those sensing modes can be distinguished clearly from one another. This is a crucial capability for the dual-capacitor sensor to be used as a multi-sensor in actuality. As discussed previously and listed in Table 1, Δ*C*_1_ and Δ*C*_2_ have a unique combination for each sensing mode in terms of their signs and magnitudes. For instance, Δ*C*_1_ and Δ*C*_2_ will be positive and one of them will be larger in magnitude than the other when the sensor is bent (Curvature sensing). Meanwhile both of them will be negative when the sensor gets just touched. Figure 4 shows the logical flow to determine what kind of physical stimulus is applied to the sensor, based on the measured capacitance changes of dual-capacitor sensor. As seen in the figure, there is no ambiguity in classifying the applied physical stimulus into curvature, pressure, strain, or touch.

**Table 1:** Summary of capacitance changes for various sensing modes.

Sensing mode	Direction	Lower capacitor (C_1_)	Upper capacitor (C_2_)	∆C_1_ vs. ∆C_2_
Curvature	convex	∆C_1_ > 0	∆C_2_ > 0	∆C_1_ < ∆C_2_
	concave	∆C_1_ > 0	∆C_2_ > 0	∆C_1_ > ∆C_2_
Pressure	upper side	∆C_1_ = 0	∆C_2_ > 0	∆C_1_ < ∆C_2_
	lower side	∆C_1_ > 0	∆C_2_ = 0	∆C_1_ > ∆C_2_
Touch	upper side	∆C_1_ < 0	∆C_2_ < 0	|∆C_1_| < |∆C_2_|
	lower side	∆C_1_ < 0	∆C_2_ < 0	|∆C_1_| > |∆C_2_|
Strain	uniaxial	∆C_1_ > 0	∆C_2_ > 0	∆C_1_ = ∆C_2_

The relative relations of upper (Δ*C*_2_) and lower (Δ*C*_1_) capacitance changes of the two capacitors in dual-capacitor sensor are uniquely distinguishable for curvature, pressure, strain, and touch sensing.

## Discussion

In conclusion, we fabricated the 5 × 5 array type of stretchable multifunctional dual-capacitor sensor, based on AgNW electrodes and PDMS insulators. Differently from other reported capacitive sensors, our dual-capacitor sensor is composed of two vertically-stacked capacitors. With the two capacitors, the sensor is capable of sensing both strength and surface-normal directionality of several physical stimuli including curvature, pressure, strain, and touch. In addition, it is confirmed that the dual-capacitor sensor can classify the physical stimulus given to it unambiguously into one of curvature, pressure, strain, and touch by comparing the capacitance changes of the two capacitors and following the logical flow designed properly. It is quite certain that our dual-capacitor sensor can enrich the information communicated in the human-machine interface. Thus, the dual-capacitor sensor is expected to be used quite extensively in various applications such as robotics, healthcare monitoring systems, and internet of things (IoT).

## Methods

### Formation of PDMS film

A PDMS (Sylgard 184, Dow Corning) film was formed by mixing the “base” and the “curing agent” with the weight ratio of 10: 1 and stirring the mixture for 5 minutes. Then, the PDMS film was stored at −4 °C for one day to remove the air bubbles residing in the film. The thickness of PDMS film was controlled easily by adjusting spin-coating speed and time. As the last step, the PDMS film free of air bubbles was cured in an oven at 100 °C for 20 minutes for hardening.

### Patterning of AgNW electrodes and contact pads

Before AgNW (0.3 wt.% of AgNW dispersed in distilled water) coating, the surface of PDMS film was activated by oxygen (O_2_) plasma treatment to convert the surface property from hydrophobic to hydrophilic, which improves the adhesion of AgNW dispersed on the PDMS surface. The O_2_ plasma treatment was performed with 100 W power for 5 minutes by using reactive ion etching (RIE, TTL Korea). In this step, we activated the PDMS surface selectively by using a shadow mask as shown in Fig. S1. With the selective activation, the AgNW electrodes could be patterned simultaneously while spin-coating the AgNW solution with no need of photolithography and etching processes. After that, we baked the sample at 110 °C for 10 minutes to remove the distilled water and leave the patterned AgNW electrodes only. Finally, the contact pads were formed by evaporating Ti (10 nm) and Au (50 nm) sequentially through another shadow mask.

### Methods of applying external stimuli

The pressure was applied by loading rectangular acrylic blocks on the dual-capacitor sensor and its magnitude was controlled by varying the number of acrylic blocks. The radius of curvature was adjusted by changing the distance between the two acrylic clamping jigs holding both ends of sensor as shown in Fig. 1c. The tensile strain was achieved by pulling both ends of sensor in the opposite directions with the two clamping jigs. Finally, the touch was done by landing the finger gently on the surface of sensor and minimizing the force exerted on it.

### Capacitance measurements

The capacitance of the dual-capacitor sensor was measured by using the Precision LCR meter (E4980A, Agilent) at the frequency of 1 MHz with a 50 mV dithering voltage controlled by our own LabView program. The LCR meter is not capable of measuring two capacitances simultaneously and hence the capacitances of the upper and lower capacitors had to be measured separately while maintaining the external stimulus given to the dual-capacitor sensor. For example, in case of curvature sensing, the capacitances of the upper and lower capacitors were measured sequentially without changing the curved shape of the sensor. The time-varying plots of the capacitance changes in touch sensing shown in Fig. 3f were obtained in the following ways: First, the capacitance change of the upper (or lower) capacitor was measured as a function of time while the touch and untouch motions were being done periodically with specified time intervals. Then, the capacitance change of the other capacitor was measured with the same time-sequential touch and untouch motions as the ones in the previous measurements. In the end, the time-varying capacitance changes of the upper and lower capacitors were plotted together in a single graph.

### Capacitances of two capacitors for dual-capacitor sensor under curvature (convex)

When the dual-capacitor is bent as shown in Fig. 1b, the capacitances of two capacitors are given as follows.

3 $$\begin{array}{l}{C}_{1}(\theta )=\frac{2\pi \,{\varepsilon }_{r}{\varepsilon }_{0}{L}_{0}}{\mathrm{ln}\,[\frac{r(\theta )+{d}_{1}(\theta )}{r(\theta )}]}\times \frac{{W}_{0}}{2\pi \,r(\theta )}\\ {C}_{2}(\theta )=\frac{2\pi \,{\varepsilon }_{r}{\varepsilon }_{0}{L}_{0}}{\mathrm{ln}\,[\frac{r(\theta )+{d}_{1}(\theta )+{d}_{2}(\theta )}{r(\theta )+{d}_{1}(\theta )}]}\times \frac{{W}_{0}}{2\pi \,r(\theta )}\end{array}$$

If assuming that the cross-sectional areas of dielectric layers are the same as those with the dual-capacitor sensor in its initial state (No bending), the following relations can be obtained for the thicknesses of dielectric layers.

4 $$\begin{array}{rcl}{W}_{0}{d}_{0} & = & [\pi {\{r(\theta )+{d}_{1}(\theta )\}}^{2}-\pi {\{r(\theta )\}}^{2}]\times \frac{{W}_{0}}{2\pi \,r(\theta )}\\  & \Rightarrow  & {d}_{1}(\theta )=-r(\theta )+\sqrt{{[r(\theta )]}^{2}+2{d}_{0}r(\theta )}\\ {W}_{0}{d}_{0} & = & [\pi {\{r(\theta )+{d}_{1}(\theta )+{d}_{2}(\theta )\}}^{2}-\pi {\{r(\theta )+{d}_{1}(\theta )\}}^{2}]\times \frac{{W}_{0}}{2\pi \,r(\theta )}\\  & \Rightarrow  & {d}_{2}(\theta )=-[r(\theta )+{d}_{1}(\theta )]+\sqrt{{[r(\theta )]}^{2}+4{d}_{0}r(\theta )}\end{array}$$

By plugging equation (4) into equation (3), the capacitances of two capacitors can be further simplified to be expressed just with the curvature radius *r*(*θ*).

5 $$\begin{array}{l}{C}_{1}(\theta )=\frac{2{\varepsilon }_{r}{\varepsilon }_{0}{L}_{0}{W}_{0}}{\mathrm{ln}[1+2{d}_{0}/r(\theta )]}\times \frac{1}{r(\theta )}\\ {C}_{2}(\theta )=\frac{2{\varepsilon }_{r}{\varepsilon }_{0}{L}_{0}{W}_{0}}{\mathrm{ln}[1+2{d}_{0}/\{r(\theta )+2{d}_{0}\}]}\times \frac{1}{r(\theta )}\end{array}$$

## Electronic supplementary material

Supplementary Information
