# Supplementary material for: Stretchable Dual-Capacitor Multi-Sensor for Touch-Curvature-Pressure-Strain Sensing
Source: Sci Rep. 2017 Sep 7;7:10854. doi: 10.1038/s41598-017-11217-w (PMC5589733; doi:10.1038/s41598-017-11217-w)
Supplement: Supplementary file 1 — Supplementary Information [file 41598_2017_11217_MOESM1_ESM.pdf]

Stretchable Dual-Capacitor Multi-Sensor for Touch-Curvature-Pressure-Strain Sensing

Hanbyul Jin[1], Sungchul Jung[2], Junhyung Kim[1], Sanghyun Heo[1], Jaeik Lim[3], Wonsang Park[3], Hye Yong Chu[3], Franklin Bien*[1], and Kibog Park*[1,2]

[1]School of Electrical and Computer Engineering, Ulsan National Institute of Science and Technology (UNIST), Ulsan 44919, Republic of Korea

[2]Department of Physics, Ulsan National Institute of Science and Technology (UNIST), Ulsan 44919, Republic of Korea

[3]Samsung Display Giheung Campus, Yongin-si, Gyeonggi-do, 17113, Republic of Korea

Corresponding Author

Correspondence and requests for materials should be addressed to Franklin Bien* (Email: bien@unist.ac.kr) and Kibog Park* (Email: kibogpark@unist.ac.kr)

The surface property of PDMS should be converted to be hydrophilic for securely attaching the AgNWs dispersed in deionized (DI) water to the surface. In order to pattern AgNW electrodes in a simple manner without photolithography and etching processes, we activate the PDMS surface selectively by using a metal shadow mask and O_2 plasma treatment. During the AgNW spin-coating process, the metal shadow mask contacted physically to the surface of PDMS film prevents AgNWs from permeating under the shadow mask. A certain portion of PDMS surface under the shadow mask could be exposed to AgNWs because of the small gap between shadow mask and PDMS surface. However, the area exposed to AgNWs can be minimal by pressing the shadow mask against the PDMS surface to induce the electrostatic glueing. Then, the DI-dispersed AgNWs will attach only on the PDMS surface activated by O_2 plasma and the clear patterns of AgNW electrodes will be obtained.

Figure S1 shows the sequences of AgNW electrode patterning by using the selective O_2 plasma treatment. For this method, we first make the metal shadow mask with the opening reflecting the intended electrode pattern exactly. During the O_2 plasma treatment, the PDMS surface exposed through the opening of shadow mask is converted to be hydrophilic from being hydrophobic. Meanwhile, the PDMS surface blocked by the shadow mask remains to be hydrophobic. In result, the AgNW electrodes can be patterned in the designed shape.

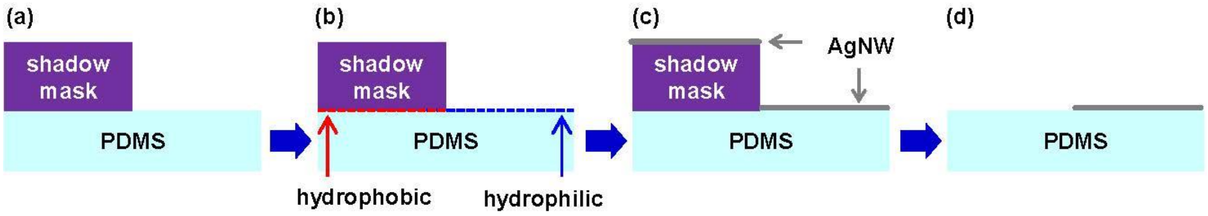

Figure S1. Schematic illustration of AgNW film pattering process using the selective O_2 plasma treatment on the area defined with a metal shadow mask

Figure S2 shows the current-voltage (I-V) curve and the optical microscope image of AgNW electrode. The AgNW electrode was patterned to have its length of ~7.5 cm and width of ~0.22 cm and it showed the line resistance of ~650 Ω in the I-V curve. The optical microscope image of AgNW electrode was obtained in the dark field mode.

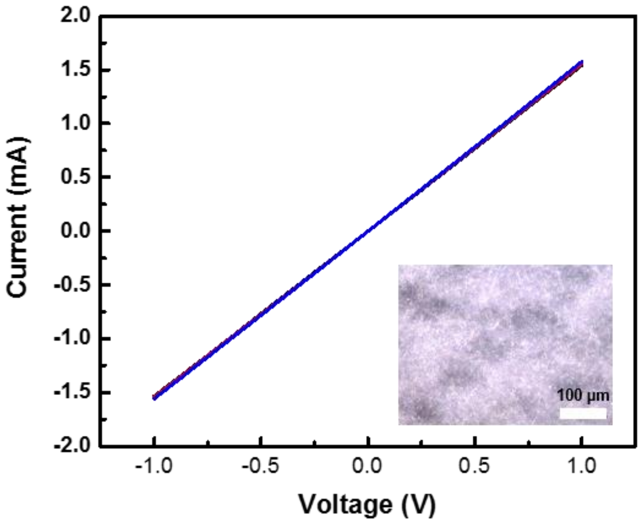

Figure S2. The current-voltage curve and the optical microscope image in the dark field mode of AgNW electrode with its length of ~7.5 cm and width of ~0.22 cm.

Figure S3 shows the cross-sectional optical microscope image of dual-capacitor multi-sensor consisting of four stacked PDMS layers including substrate, lower insulating layer, upper insulating layer, and protective layer. The lower and upper insulating layers have similar thickness of ~400 μm and the stacked PDMS layers are found to be adhered firmly to each other. The AgNW electrodes were patterned on the substrate, lower insulating layer, and upper insulating layer. However, they are not visible in the cross-sectional optical microscope image since they are so thin (nm-scale).

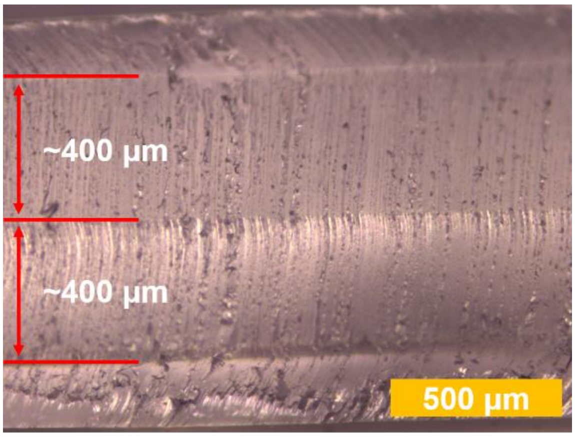

Figure S3. The cross-sectional optical microscope image of dual-capacitor multi-sensor.

The stretchability test of dual-capacitor sensor was conducted by checking its capacitance change after multiple stretching(20%)-releasing cycles. The capacitance of dual-capacitor sensor was measured every 100 cycles in the released state, and this test was performed up to 1,000 cycles. As shown in Fig. S4, the capacitance changes of lower (C_1) and upper (C_2) capacitors are always within 2 % regardless of the cycle number. This indicates that our dual-capacitor sensor has excellent robustness for continuous stretching-releasing operations.

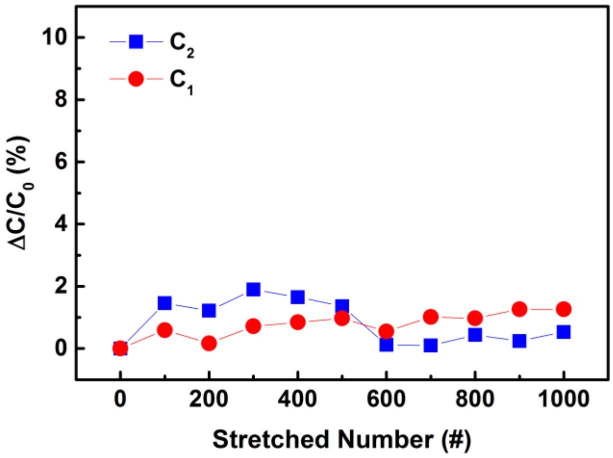

Figure S4. The relative capacitance change depending on the number of stretching-releasing cycles

Figure S5 shows the time-dependent capacitance changes of dual-capacitor sensor while it is stretched and released with the strain increasing gradually. Once the sensor is released from stretching, the capacitance is found to return to its initial value regardless of the stretching strength. This indicates that no residual strain remains after the stretching is removed.

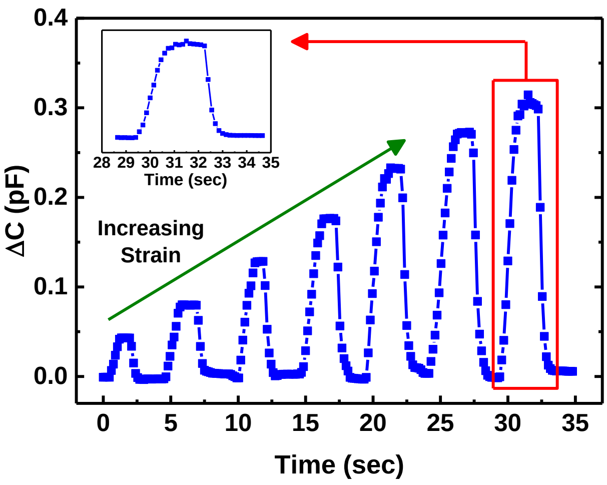

Figure S5. The time-dependent capacitance change while the dual-capacitor sensor is under repeated stretching-releasing operations with the strain increasing gradually.
